# Impact Loading and Locomotor-Respiratory Coordination Significantly Influence Breathing Dynamics in Running Humans

**DOI:** 10.1371/journal.pone.0070752

**Published:** 2013-08-12

**Authors:** Monica A. Daley, Dennis M. Bramble, David R. Carrier

**Affiliations:** 1 Department of Comparative Biomedical Sciences, Royal Veterinary College, Hatfield, Hertfordshire, United Kingdom; 2 Department of Biology, University of Utah, Salt Lake City, Utah, United States of America; The University of Queensland, Australia

## Abstract

Locomotor-respiratory coupling (LRC), phase-locking between breathing and stepping rhythms, occurs in many vertebrates. When quadrupedal mammals gallop, 1∶1 stride per breath coupling is necessitated by pronounced mechanical interactions between locomotion and ventilation. Humans show more flexibility in breathing patterns during locomotion, using LRC ratios of 2∶1, 2.5∶1, 3∶1, or 4∶1 and sometimes no coupling. Previous studies provide conflicting evidence on the mechanical significance of LRC in running humans. Some studies suggest LRC improves breathing efficiency, but others suggest LRC is mechanically insignificant because ‘step-driven flows’ (ventilatory flows attributable to step-induced forces) contribute a negligible fraction of tidal volume. Yet, although step-driven flows are brief, they cause large fluctuations in ventilatory flow. Here we test the hypothesis that running humans use LRC to minimize antagonistic effects of step-driven flows on breathing. We measured locomotor-ventilatory dynamics in 14 subjects running at a self-selected speed (2.6±0.1 ms^−1^) and compared breathing dynamics in their naturally ‘preferred’ and ‘avoided’ entrainment patterns. Step-driven flows occurred at 1-2X step frequency with peak magnitudes of 0.97±0.45 Ls^−1^ (mean ±S.D). Step-driven flows varied depending on ventilatory state (high *versus* low lung volume), suggesting state-dependent changes in compliance and damping of thoraco-abdominal tissues. Subjects naturally preferred LRC patterns that minimized antagonistic interactions and aligned ventilatory transitions with assistive phases of the step. Ventilatory transitions initiated in ‘preferred’ phases within the step cycle occurred 2x faster than those in ‘avoided’ phases. We hypothesize that humans coordinate breathing and locomotion to minimize antagonistic loading of respiratory muscles, reduce work of breathing and minimize rate of fatigue. Future work could address the potential consequences of locomotor-ventilatory interactions for elite endurance athletes and individuals who are overweight or obese, populations in which respiratory muscle fatigue can be limiting.

## Introduction

Effective ventilation is essential for sustained animal locomotion. For many animals, including birds and mammals, this requires integrating movement and breathing so that inspiration and expiration occur during mechanically compatible periods of the locomotor cycle. Several direct mechanical links between locomotion and ventilation necessitate integration [1], [2], [3], [4]. Firstly, sagittal bending of the trunk assists forward progression during locomotion in quadrupeds, and creates a ‘bellows’ effect, altering the pressure and volume of the abdomen and thorax. Additionally, impact loads induce inertial motions of soft-tissues (viscera, adipose), creating a ‘visceral piston’ effect, pulling and pushing on the diaphragm and body wall muscles (abdominals, intercostals) and altering thoraco-abdominal pressures. Finally, many axial muscles of terrestrial vertebrates contribute to both breathing and locomotion [5], [6], [7], [8], [9], [10]. Indeed, many ‘ventilatory muscles’ cease respiratory action and become entrained with the locomotor cycle during running in lizards [5], [6], birds [7] and dogs [8], [9]. As a result of these factors, active inspiration is most compatible with a specific and different phase of the locomotor cycle than active expiration.

Locomotor-respiratory coupling (LRC) refers to phase locking of running and breathing so that the same number of steps occur during each breath, and has been observed in numerous vertebrates, including birds, dogs, hares, horses, wallabies and humans [2], [3], [11], [12], [13]. LRC is a form of entrainment, in which the two rhythmic activities with different frequencies become phase-locked due to mechanical and neural interactions. LRC has been suggested to have a number of important physiological effects. These include reducing the energy cost of breathing [14], [15], [16], [17], minimizing conflict in muscles that contribute to both functions [6], [9], body stabilization during motion [11], [18], and enabling trunk bending and inertial movements of soft-tissues to augment pumping of air in and out of the lungs [2], [19].

Whereas most galloping mammals exhibit 1∶1 (strides/breath) coupling, humans demonstrate more flexibility in breathing patterns during locomotion. Humans frequently use LRC ratios of 2∶1, 2.5∶1, 3∶1, or 4∶1 and sometimes lack entrainment altogether, using independent breathing and stepping frequencies [2], [20], [21], [22], [23]. Variance in LRC pattern is observed both within and across individuals [14], [21], [22], [23], [24]. This flexibility in human breathing patterns relative to locomotion most likely relates to an upright posture and striding bipedal gait. Unlike quadrupeds, the sagittal bending of the back does not assist forward progression and the forelimbs are not subjected to direct weight-bearing and impact loading. The forces transmitted through the thorax, abdomen and rib cage are consequently smaller, reducing mechanical interactions between locomotion and ventilation. Ground birds, which are also bipedal, exhibit similar flexibility as humans in locomotor-ventilatory coupling patterns [3], [17]. Bipedal posture may reduce the mechanical and neuromuscular conflicts that constrain quadrupeds to a 1∶1 LRC pattern [8], [9].

Does this greater flexibility suggest that breathing and running are mechanically independent in humans? The answer remains controversial. Humans do often exhibit sustained ventilatory entrainment to locomotor forces [2], [21], [22]. Furthermore, evidence suggests coupling improves breathing efficiency in some circumstances [2], [15], [24]. On the other hand, humans also couple breathing frequency with low impact, non-locomotor movements such as finger and arm tracking [25], [26]. Thus, large mechanical interactions are not required to induce rhythmic coupling. Theoretically, human LRC during running could arise entirely from neural interactions.

The overall ventilatory effect of locomotor-ventilatory interactions likely depends on a number of neural and mechanical factors. The primary mechanical factors in mammalian locomotor-ventilatory interactions are thoracic loading and inertial displacement of the soft-tissues associated with body accelerations [2], [12], [19], [20], [27], [28]. In humans, impact loading of the body at footstrike is likely to cause compression of the thorax and downward displacement of the abdominal viscera. Arm swinging is also likely to exert a compression load on the thorax during early to mid-stance [29]. These mechanical actions can alter respiratory flow by changing thoraco-abdominal volumes and pressures. Inertial displacement of the abdominal viscera would tend to pull or push on the respiratory diaphragm through its direct attachment to the liver. The relative magnitude of the effects on the rib cage and diaphragm likely depends on the mass of the abdominal organs, the distribution of other soft-tissues, and the vertical accelerations of the body. Body wall muscles including the intercostals and abdominals could act to resist these effects [30], [31]. Additionally, the rhythmic activity of intercostal and abdominal muscles is entrained by afferents from the arms and legs, facilitating neural coupling [32], [33]. The ventilatory outcome of these neuro-mechanical interactions likely depends on the relative timing and magnitude of the mechanical factors (thorax compression and visceral displacement) and the rhythmic activation of body wall muscles.

To address whether locomotor-ventilatory interactions are mechanically significant in running humans, Banzett and colleagues [20] measured ‘step-driven flow’ in humans running on a treadmill. ‘Step-driven flow’ is ventilatory flow and resulting volumes attributable to locomotor-induced forces, once the primary ventilatory pattern is removed (through either ensemble averaging or filtering – see methods). Banzett and colleagues [20] found the volumes attributable to step-driven flows to be a negligible fraction of tidal volume, ∼1–2% tidal volume per step. Consequently, they concluded that locomotion has no meaningful mechanical influence on breathing in humans [20]. Following publication of this finding, there has been relatively little research on the mechanical consequences of locomotor-ventilatory interactions in running humans.

Yet, there are reasons to revisit the potential mechanical significance of locomotor-ventilatory interactions in humans. In the study by Banzett and colleagues, the subjects lightly rested their hands on side rails while jogging on the treadmill [20]. Arm swinging contributes to compression loads on the thorax during locomotion, and to control of body and head rotation [29], [34]. The restriction of natural arm motion may have reduced mechanical interactions between locomotion and ventilation. Furthermore, although the volumes attributed to step-driven flows amounted to only 1–2% of tidal volume per step, Banzett and colleagues measured large transient flows associated with footstrike [20]. These step-driven flows lasted only a small fraction of the ventilatory cycle, but exhibited peak magnitudes of 1 Ls^−1^. Although brief, step-driven flows reach a large fraction of concurrent ventilatory flow, sometimes resulting in a brief mid-breath reversal in flow (from inspiration to expiration or vice-versa). We suggest that these transient flows have potential to influence breathing dynamics and ventilatory muscle loading without directly driving large ventilatory volumes.

Here we test the hypothesis that humans use LRC to minimize antagonistic effects of step-driven flows on breathing dynamics. Due the natural variability in coupling patterns in running humans [14], [21], [22], [23], [24], it is possible to compare breathing dynamics when individuals use their own ‘preferred’ and ‘avoided’ entrainment patterns. We analyze locomotor-ventilatory interactions in fourteen physically fit subjects running on a treadmill at a moderate self-selected speed (2.6±0.1 ms^−1^) with natural arm motion. Self-selected speed is likely to be close to energetically optimal speed [35]. Running at speeds above preferred speeds would increase both impact loads and ventilatory demand, increasing the potential for antagonistic locomotor-ventilatory interactions. We therefore consider self-selected speed to provide a conservative test of the hypothesis that humans use LRC to minimize antagonistic locomotor-ventilatory dynamics.

## Materials and Methods

### Ethics Statement

The Institutional Review Board of University of Utah approved the protocols and informed consent documents, under IRB #12361, and all subjects gave written informed consent.

### Subjects and protocol

Fourteen adult subjects volunteered for this study (9 females 5 males; age 36±2 years, mass 65.1±2.8 kg, height 1.72±0.02 m). Seven of the subjects were recreational runners who regularly ran at least 20 miles per week, and seven were physically fit non-runners (Table 1). This sampling was designed to obtain a broad representation of human locomotor-ventilatory patterns, not to statistically compare trained and non-trained runners, which has been previously studied by others [23], [24]. Participants ran at a steady speed on a treadmill, using natural arm motion. After an initial practice session, subjects were allowed to select their preferred speed, with the instructions to run at a comfortable pace that they could sustain for 30 minutes. They warmed up for 5 minutes and then continued to run for 5 minutes while we recorded running and breathing patterns.

**Table 1:** Subject data.

Subject	Gender	Age (yrs)	Mass (kg)	Height (m)	Speed (ms)	Training	Entrainment	Coupling Ratio (s)
1	F	39	54.0	1.57	2.4	Casual	Coupled, vp,vr*	2:1, 3:1
2	F	41	59.4	1.73	2.4	Nonrunner	Coupled	2:1
3	M	35	72.5	1.75	2.9	Nonrunner	Coupled, vr*	3:1, 3.5:1
4	M	43	76.2	1.83	2.4	Nonrunner	Uncoupled*	
5	F	43	60.8	1.73	2.6	Casual	Coupled, vp*	2:1
6	F	23	59.0	1.65	2.4	Nonrunner	Coupled, vp*	2:1
7	M	26	86.2	1.78	2.4	Nonrunner	Coupled, vp,vr*	2:1, 2.5:1, 3:1
8	F	41	51.9	1.67	2.4	Casual	Coupled, vp*	2.5:1
9	F	28	64.4	1.70	2.4	Nonrunner	Uncoupled*	
10	M	49	71.7	1.78	2.8	Trained	Coupled, vr *	2:1, 3.5:1
11	F	47	62.6	1.65	2.4	Trained	Coupled, vp,vr*	2:1,3:1
12	F	27	59.0	1.75	3.4	Trained	Coupled,vp*	2:1
13	F	25	54.5	1.60	3.4	Trained	Coupled	2:1
14	M	31	79.4	1.83	2.3	Nonrunner	Uncoupled*	
	mean	36	65.1	1.72	2.6			
	s.e.m.	2	2.8	0.02	0.1			

* Ensemble average calculated for this subject.

Coupling abbreviations: vr  =  variable coupling ratio. vp  =  variable coupling phase relationship.

### Data collection

We tracked the step cycle by measuring acceleration at the top of the head using an Endevco microtron accelerometer (model 7290A-10). The subjects wore a lightweight facemask equipped with two-way non-rebreathing valves to measure inspiratory and expiratory flow separately (Hans Rudoph Inc., Kansas City, MO, USA). Each valve was connected by tubing (34.2 mm ID; Hans Rudolph, Inc.) to screen pneumotachs (model 4813, Hans Rudolph Inc.), with a calibrated flow range of 0–800 liters per minute. The pressure ports of the screen pneumotachs were attached to a differential pressure transducer (Omega Engineering, Stamford CT, USA). To prevent motion artifact, the tubing and pneumotachs were secured to a small backpack worn by the subject, fitted with both waist and shoulder straps. Flow was calibrated by recording the output voltage from a known volume of air, using a 3 liter calibration syringe (Hans Rudolph Inc.).

The signals were amplified 10x (model P122, Grass Inc., West Warwick RI, USA), sampled at 200 Hz and digitally recorded onto computer using the MP100 data acquisition system with AcqKnowledge software (BIOPAC Systems Inc., Goleta, CA, USA.). Before analysis, data were low pass filtered at 30 Hz using a zero-phase 6^th^ order Butterworth filter in Matlab (butter and filtfilt functions; Matlab release 13). These filter settings were selected because they were found to effectively remove noise while retaining higher frequency signal characteristics such as peaks associated with footstrike impact.

### Calculation of average step-driven flow

To enable comparison to previous findings, we calculate average step-driven flow using similar analytical methods of Banzett and colleagues [20]. We calculated an ensemble average (EA) of ventilatory flow relative to the step cycle for each individual, including at least 50 steps per subject [20]. The EA technique samples flow segments distributed equally through the ventilatory cycle, to quantify step-driven flow independent of ventilatory phase. We calculated this EA for the 12 out of 14 subjects who exhibited varying phase of entrainment between running and breathing cycles (Table 1). The resulting EA trace of step-driven flow was integrated over time to obtain the locomotor driven volume (LDV). Expiratory (positive) and inspiratory (negative) volumes were integrated separately to assess the relative magnitude of each. The LDV was normalized by the total concurrent ventilatory volume (V_tot_) over the same time periods as the EA flow segments, to obtain a value as a percentage of concurrent ventilatory flow. Note that our calculation of LDV differs slightly from that used by Banzett and colleagues, who reported the fractional contribution to tidal volume *per step*; whereas we report the total fractional contribution to ventilation. Humans typically use coupling ratios of 2∶1 and higher (e.g. [2], [20], [21], [22], [23]), taking multiple steps in each breath cycle; consequently, a *per step* value does not provide a complete account of the total magnitude of locomotor-driven ventilation.

A potential source of error in the calculated LDV is flow artifact caused by motion of the tubes during running. As mentioned earlier, we minimized this by securing the tubes to a lightweight backpack worn by the subject. To quantify the error associated with any remaining motion, we calculated an ‘artifact LDV’ for 5 subjects. We recorded inspiration and expiration in separate channels, causing each channel to have a baseline of zero during the opposing ventilatory half-cycle. During this period, the only flow measured in that channel was small magnitude, high frequency artifact flow due to tube motion. To quantify this, we calculated a value of LDV using the same method described above, except that the flow segments in the ensemble average were obtained from the baseline of the inactive ventilation channel. This method resulted in an artifact LDV value of 0.7±0.1% of V_tot_ (mean ± s.e.m.) during control running trials, with a worst case artifact magnitude of 1% V_tot_.

### Testing for variation in step-driven flow with ventilatory phase

**Figure 1:**
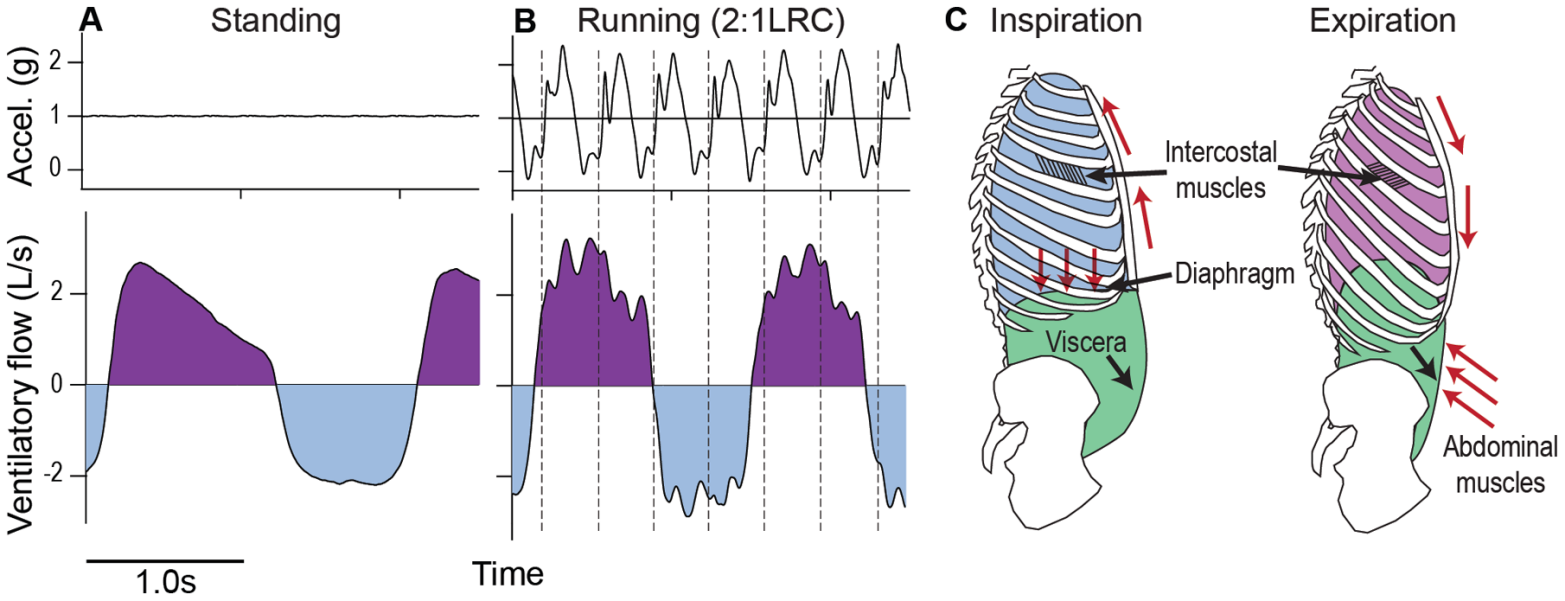
Locomotor-ventilatory interactions. (**A–B**) Typical head acceleration (top) and ventilatory flow (bottom: expiration positive, inspiration negative) during quiet standing (**A**), and moderate speed treadmill running (**B**). Note the high frequency oscillations in ventilatory flow during running. (**C**) Schematic illustration of the ‘visceral-piston’ model for human locomotor-ventilatory interactions. Red arrows indicate muscle actions during inspiration and expiration.

The visceral piston hypothesis predicts that the effect of locomotor forces on flow depends on the phase of the breath cycle in which the step occurs [2] (Fig. 1C). Deceleration of the body at footstrike likely has two primary mechanical effects: 1) compression of the thorax, and 2) viscoelastic bounce and rebound of the guts [20], [28]. Contraction of respiratory muscles and inflation of the lungs differentially alters thoracic and abdominal stiffness [36]. Locomotor forces are likely to have an enhanced expiratory effect if they occur when the abdominal muscles are being recruited for forced expiration, because these muscles resist the descent of the guts (Fig. 1C).

The ensemble average technique cannot reveal this effect of ventilatory phase because it averages step-driven flow across all phases. Therefore, we also compared step-driven ventilation at different phases of the breathing cycle, in bins of 20% of maximum tidal volume (V_max_) during inspiration and expiration. From the ensemble averaged data, we observed that step related oscillations in flow occurred at step frequency and the 1^st^ and 2^nd^ harmonic of step frequency. We high-pass filtered the flow signal to remove the primary breathing pattern, so that only flows associated with the step frequency or higher remained in the signal. We used a zero-phase digital Butterworth filter (‘butter’ and ‘filtfilt’ functions in Matlab version 6.5), with the cutoff frequency and filter order specified using the Matlab function ‘buttord’ to create a filter that lost no more than 1% of the signal greater than or equal to step frequency and attenuated 99% of the signal below half the step frequency. We then calculated an average ± s.e.m of step-driven flow for all steps occurring within each volume bin of the ventilatory cycle.

### Analysis of entrainment patterns

We used phase analysis to examine the locomotor-ventilatory coupling patterns. The timing of each inspiratory and expiratory transition was calculated relative to the step cycle. The beginning of footstrike was defined as zero, and the phase of each ventilatory transition was expressed in degrees between 0 and 360 or as a fraction of the step cycle by dividing the phase angle by 360. The step cycle was divided into 20 bins (18 degrees or 5% of the step cycle each), and the frequency of inspiratory and expiratory transitions was calculated for each bin. If breaths and steps occur randomly with respect to each other, the distribution of ventilatory transition events should not significantly differ from a uniform circular distribution. For each subject whose ventilatory transitions significantly differed from a uniform circular distribution (see *Statistics*), we determined the ‘preferred’ phase for inspiratory and expiratory initiation as the bin in which each of these events occurred most frequently. Likewise, we determined ‘avoided’ phase as the bin in which each transition event occurred least frequently.

### Ventilatory transition times

To quantify the time required for transition between ventilatory half cycles (inspiration and expiration), we measured the time between 50% peak flows (T50). The expiratory T50 is the time from 50% peak inspiration to 50% peak expiration. Likewise, the inspiratory T50 is the same measure between 50% peak expiration and 50% peak inspiration. We compared T50 values between ‘preferred’ and ‘avoided’ phase bins, described above. If multiple bins were tied for ‘preferred’ or ‘avoided’, we took the average of the tied bins.

### Statistics

Kuiper’s test of circular uniformity was used to test whether inspiratory and expiratory initiation events were distributed randomly with respect to the step cycle. For subjects with ventilatory transitions distributed non-uniformly relative to the step cycle, a Friedman nonparametric repeated measures ANOVA test was used to compare ventilatory transition times (T50) between preferred and avoided phases of step cycle, with posthoc pairwise comparisons using Dunn’s Multiple Comparisons.

## Results

### Step-driven oscillations in flow

**Figure 2:**
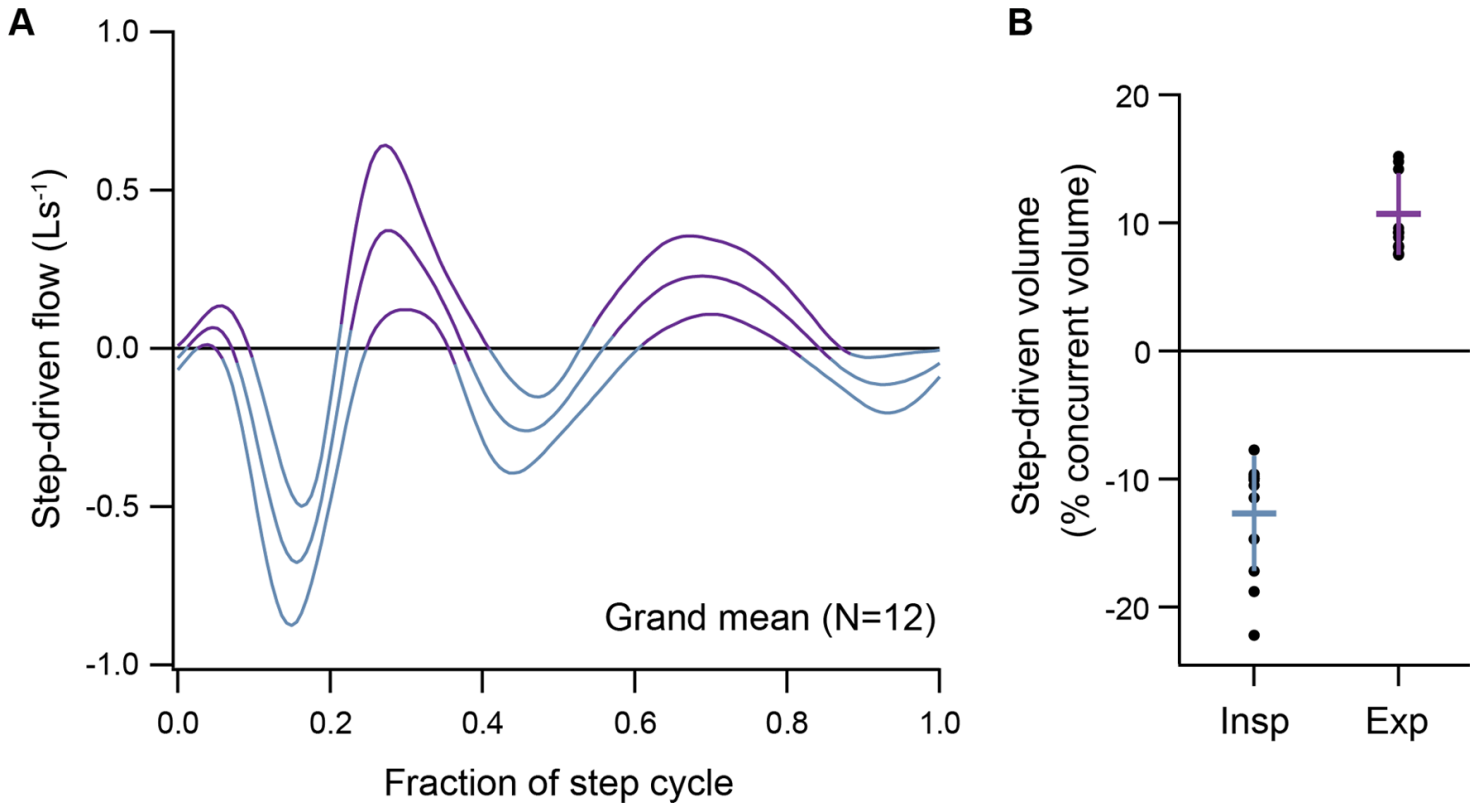
Step-driven ventilatory flows and volumes. (**A**) Grand mean of step-driven ventilatory flow across subjects (mean ± 95% confidence interval for 12 subjects, see also individual examples in Fig. 5). Expiration is positive. (**B**) Mean ± SD of step-driven volume as a percentage of total concurrent ventilatory volume (V_tot_), during level moderate speed running (N = 12, black dots show data from individuals). Step-driven flow and volume data included here are from subjects with variation in the phase locking between steps and breaths (**Table** **1**).

**Figure 3:**
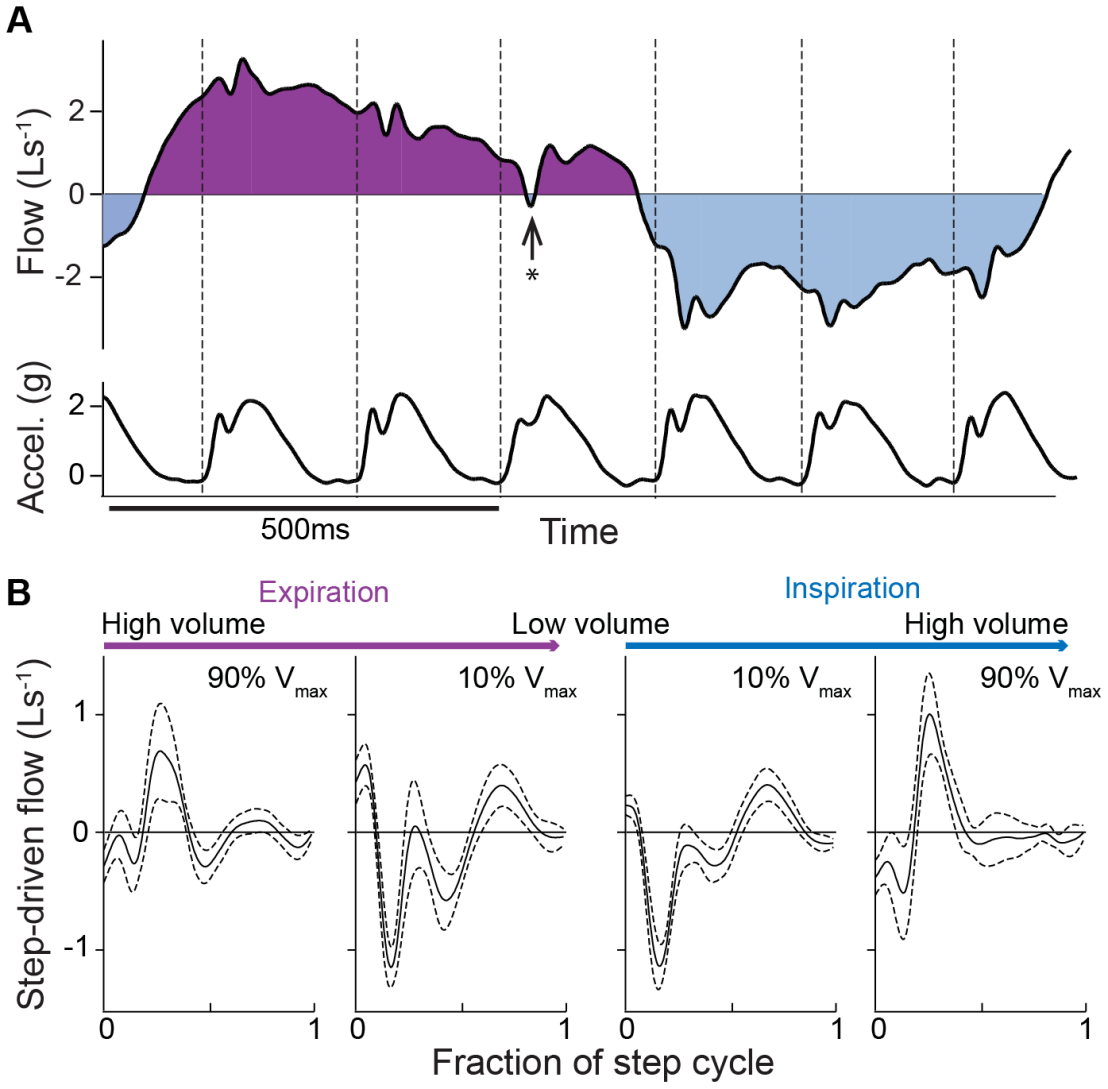
Step driven flow depends on ventilatory phase. **(A)** Variation in the magnitude of step-driven flows is apparent, particularly during low frequency breathing, as shown here during 3∶1 (strides per breath) coupling. Note the reversal in flow in late expiration (asterisk). Dashed vertical lines indicate footstrike events (data from subject 3). (**B**) Average step-driven flow (means ±95%CI) for a representative subjective at four points in the ventilatory cycle: 1) early expiration, high lung volume (90% V_max_), 2) late expiration, low lung volume (10% V_max_), 3) early inspiration, low lung volume (10% V_max_) and 4) late inspiration, high lung volume (90% V_max_).

All subjects exhibited high frequency, step-driven oscillations in flow (Fig. 1B). An inspiratory pulse occurred immediately after footstrike, followed by an expiratory pulse as the body approached peak acceleration (Fig. 2). The associated inspiratory and expiratory volumes amounted to −12.7±4.5% (mean ±S.D) and 10.7±3.2%, respectively, of the concurrent ventilatory volume (Fig. 2; Table 2), with peak flow magnitude averaging 0.97±0.45 Ls^−1^ across individuals. In some cases, these step-driven flows were large enough to cause transient reversals in flow (Fig. 3A).

**Table 2:** Step-driven ventilatory volume, as a fraction of concurrent volume, during inspiration and expiration.

Subject	Insp	Exp
1	−17.2%	15.2%
**3**	−9.8%	9.3%
**4**	−7.7%	7.6%
**5**	−10.5%	7.5%
**6**	−9.6%	9.2%
**7**	−11.5%	8.2%
**8**	−9.9%	8.1%
**9**	−10.1%	9.6%
**10**	−14.7%	14.8%
**11**	−18.8%	14.2%
**12**	−22.2%	15.4%
**14**	−9.9%	8.9%
mean	−**12.7%**	**10.7%**
s.d.	**4.5%**	**3.2%**

### Locomotor-ventilatory interactions and entrainment

**Figure 4:**
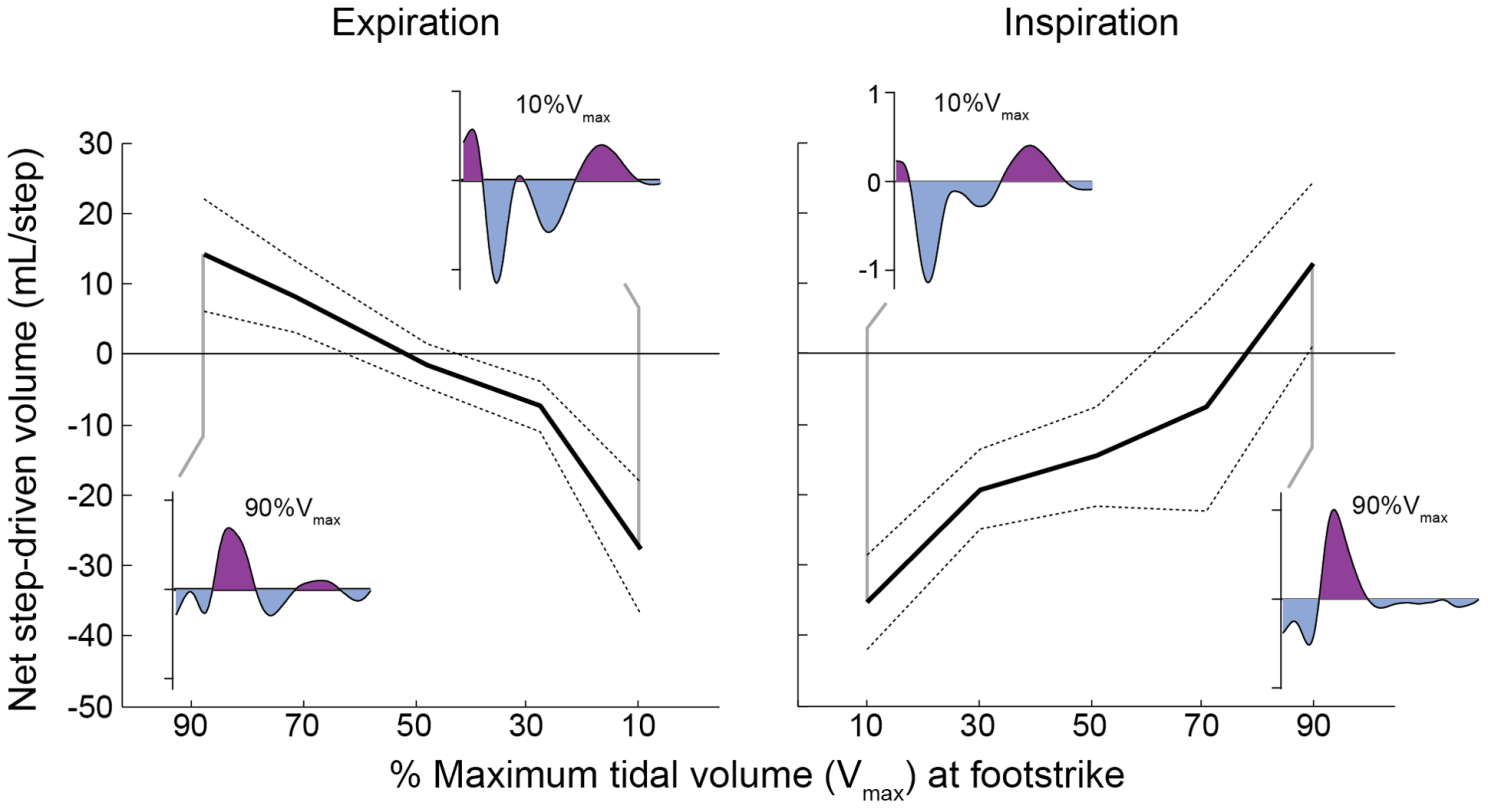
The net effect of step-driven ventilation shifts from synergistic to antagonistic in each ventilatory half-cycle. We show net step-driven volumes (in milliliters per step) as a function of the tidal volume at the time of footstrike during inspiration (left) and expiration (right), averaged across individuals (mean ± 95% CI, N = 12). At low lung volumes, the inspiratory pulse at footstrike is larger, leading to a net inspiratory effect. At high lung volumes, a larger expiratory pulse occurs, leading to a net expiratory effect.

Although the step-driven inspiratory and expiratory volumes were equal on average (Fig. 2), the effect of locomotor acceleration on flow varied considerably with ventilatory phase (Fig. 3). Across individuals, we observed a significant relationship between tidal volume at footstrike and the net step-driven volume (Fig. 4). Steps occurring in late expiration (low lung volume) tend to have a net inspiratory effect. Similarly, steps occurring in late inspiration (high lung volume) tend to have a net expiratory effect. Thus, the net effect of step-driven flow is synergistic early in the ventilatory half-cycle and antagonistic late in each ventilatory half cycle. Flow reversals occur most often in late expiration during slow, deep breaths and coupling with ratios greater than 2.5∶1 strides per breath (Fig. 3A). However, most runners avoided these flow reversals because they breathed more frequently, closer to a 2∶1 stride per breath rhythm.

**Figure 5:**
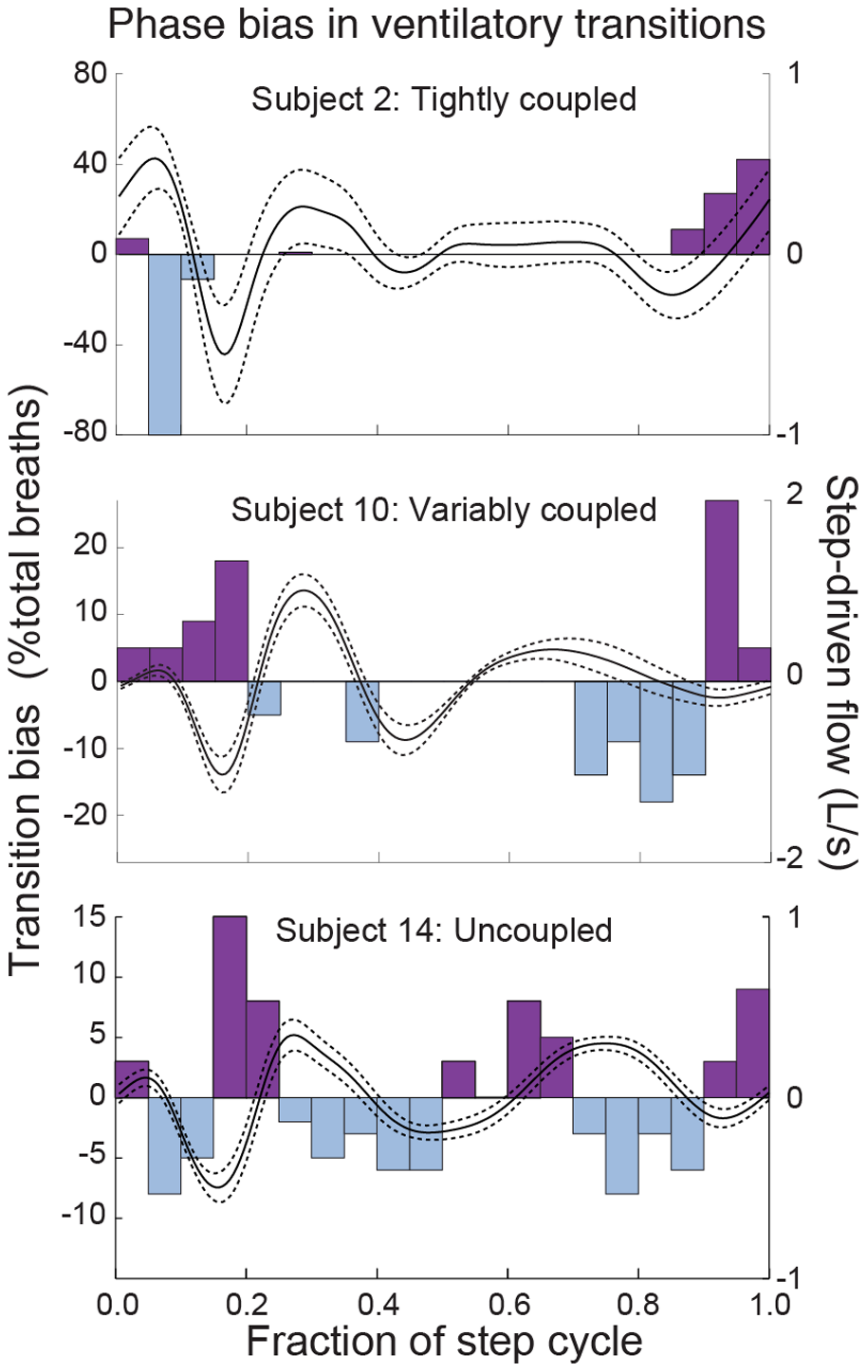
Subjects prefer to initiate ventilatory transitions at phases that assist rather than imped flow. The distribution of ventilatory transitions relative to the step cycle is non-uniform (Table 3). Here we show the net bias in ventilatory transitions relative to step cycle (left axis and bars), with transitions to expiration as positive, transition to inspiration as negative. The ‘net bias’ value is the number of expiratory transitions minus the number of inspiratory transitions in each phase bin. Step-driven flow is overlaid for reference (right axis and lines). Data from 3 individuals illustrates typical variation between strongly coupled (**A**), variably coupled (**B**) and uncoupled (**C**) subjects. Although variation exists, the timing of transitions is clearly non-random, and exhibits some correspondence to the step-driven flow.

Runners varied in whether or how tightly they entrained breathing and stepping rhythm (Table 1). Most (11 of 14) subjects exhibited periods of fixed-ratio coupling between stepping and breathing rhythms, with 2∶1 strides per breath as the most common pattern. Two of these subjects exhibited strict phase-locked coupling with a single coupling ratio, whereas the remaining 9 subjects exhibited varied phase or switching among multiple coupling ratios (Table 1). Despite variation in coupling, 13 of 14 subjects had significantly non-uniform distributions of ventilatory transitions relative to the step cycle (inspiratory, expiratory or both; Table 3). Preferred transition phases tended to correspond to regions of the step cycle that mechanically assisted ventilation, or at least, did not impede it (Fig. 5).

**Table 3:** Mean phase and dispersion (in degrees) of ventilatory transitions relative to the step cycle.

	Phase relative to step cycle (degrees)					
Subject		Inspiration		Expiration		
	N	mean	disp	mean	disp	
1	57	296*	13.0	121*	6.2	
2	72	32*	0.1	352*	0.2	
3	48	345*	0.4	285*	0.6	
†4	70	286	16.0	282	11.6	
5	86	176*	0.9	164*	3.0	
6	82	28*	0.5	313*	1.2	
7	62	297*	1.1	273*	9.5	
8	87	325*	0.8	255*	1.1	
9	55	203	99.0	334*	0.9	
10	35	296*	0.5	356*	0.5	
11	79	153*	6.2	154*	1.8	
12	90	282*	1.2	150*	8.2	
13	87	308*	0.4	212*	0.4	
14	62	324	26.0	17*	13.6	

* Indicates significant difference from uniform circular distribution based on Kuiper’s test.

† Indicates subject with no statistical evidence of transition entrainment in inspiration or expiration.

**Figure 6:**
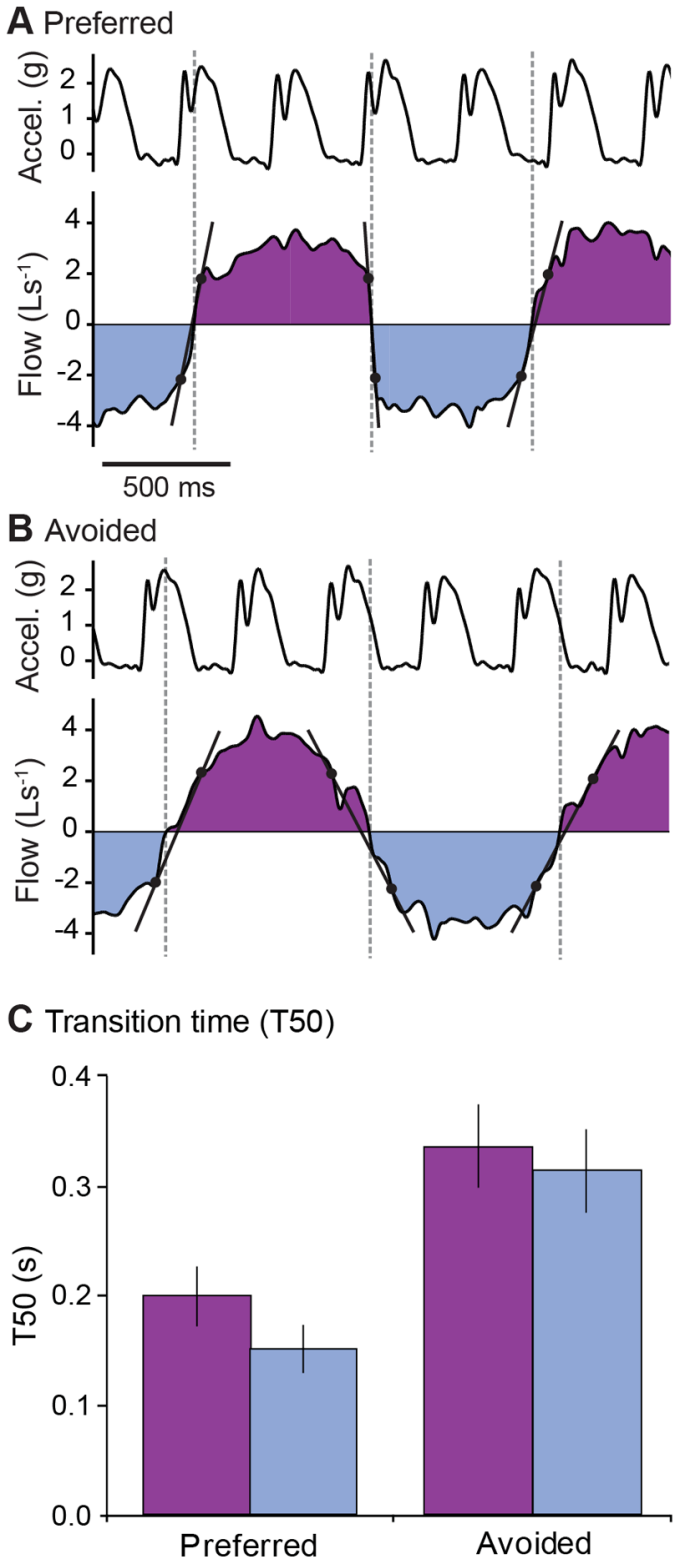
Phasing of steps relative to breaths has a significant effect on the duration of ventilatory transitions. **(A)** Examples of ventilatory transitions timed with preferred and assistive phases of the step cycle, facilitating rapid transitions, and (**B**) timed with an avoided and antagonistic phases of the step cycle. Dashed vertical lines indicate zero-crossings of ventilatory transitions. The transition durations (T50) are calculated between the black dots indicating the times of 50% peak flows. **(C)** Transition times averaged across individuals (mean ± s.e.m), comparing breaths at ‘avoided’ and ‘preferred’ phase relationships. Preferred refers to the most used phase bin (e.g., see Fig. 5), and ‘avoided’ refers to the least used phase bin that was represented in the data.

Although step-driven flows are brief, the phasing of transitions relative to the step cycle does substantially influence breathing dynamics. Whether coupled or not, most runners avoided reversals in flow by using a breathing frequency near a 2∶1 stride per breath ratio and timing ventilatory transitions to coincide favorably with step-driven flow (Fig. 5). Furthermore, the phasing between steps and breath transitions influenced the time required for ventilatory transitions. When runners coordinate the transitions to occur in phases of the step cycle that promote flow, transitions occur 2X more rapidly (Fig. 6). When compared across all uncoupled or variably coupled runners (for which ‘avoided’ transition data were available), ventilatory transitions in ‘preferred’ phase relationships with the step cycle occurred more rapidly than those in ‘avoided’ phases (Fig. 6). The difference between preferred and avoided transition T50 values was statistically significant (p = 0.0002 for Freidman Test, p<0.05 Dunn’s Multiple Comparisons).

## Discussion

The goal of this study was to examine whether the mechanical interaction between running and breathing in humans is large enough to be physiologically important. We measured the ventilatory flows and volumes attributable to step-driven flows during running at a moderate self-selected speed. The timing of step-driven flows is consistent with the visceral piston hypothesis for locomotor-ventilatory interactions in humans [2], [19], [20], [27], [28]. Step-driven ventilation averaged around 10–12% of total ventilatory volume with flow magnitudes around 1 Ls^−1^. We found that step-driven flow dynamics varied depending on ventilatory state (high *versus* low lung volume), suggesting phase-dependent changes in compliance and damping of thoraco-abdominal tissues. We also discovered that the timing of impact loading relative to the ventilatory cycle significantly influenced breathing dynamics. Ventilatory transitions initiated in preferred (assistive) phases of the step cycle occurred 2x faster than those in avoided (antagonistic) phases. These findings suggest a physiologically significant mechanical interaction exists between locomotion and ventilation in humans.

The step-driven ventilatory volumes we measured appear to be larger on average than those reported previously [20]. Here, step-driven volumes amounted to 10–12% of total ventilation, which amounts to 2.5–3.0% tidal volume *per step* during a 2∶1 step per breath rhythm. Banzett and colleagues reported a value of 1–2% tidal volume *per step*. Nonetheless, the timing of peak step-driven flow was consistent with Banzett and colleagues. The difference in magnitude could be explained by a difference in arm motion between the two studies. In the earlier study, the subjects lightly rested their hands on side rails, whereas in the current study the subjects were allowed to move their arms naturally while running. Both step-induced thoracic loading and inertial displacement of abdominal viscera likely contribute to locomotor-ventilatory interactions while running [2], [12], [19], [20], [27]. Minimizing the effects of arm loading on thoracic compression during running could reduce both the mechanical and neural interactions between locomotion and ventilation [29], [32], [33], [37]. Nonetheless, we do find a similar pattern, and although the total step-driven volumes are small on a *per step* basis, they do have a significant mechanical influence on breathing dynamics.

### What is the physiological significance of locomotor-ventilatory entrainment in humans?

The frequency of step related flows is too high relative to breath frequency to allow direct coupling to drive ventilation (Figs. 1, 2). Yet, our data reveal that runners prefer to time ventilatory transitions to periods in the step cycle that assist rather than impede flow (Fig. 5), and this timing significantly reduces the time required for transitions (Fig. 6). These data suggest that step-driven flows have potential to influence the work of respiratory muscles, because they significantly influence breathing dynamics, particularly at ventilatory transitions (expiration to inspiration and *vice versa*). Appropriate coordination of stepping and breathing rhythm may act to minimize antagonistic loading of the respiratory muscles caused by motions of the abdomen and chest wall. Although locomotor-ventilatory coupling is more flexible in humans than in quadrupedal mammals [2], [3], [11], [12], [13], [22], coupling in humans may reduce conflicting demands placed on the diaphragm and body wall muscles (abdominal muscles and intercostals).

The 2∶1 LRC pattern preferred by human runners may also reflect optimization to minimize antagonistic loading of respiratory muscles. Humans prefer a 2∶1 ratio across a wide range of sustainable running speeds (e.g. [2], [20], [21], [22], [23]). A recent study by O’Holloran and colleagues found the highest flexibility in LRC patterns at preferred stride frequency, when energy cost is minimal. At stride frequencies above or below preferred, energy demand sharply increases and subjects exhibit a stronger preference for 2∶1 coupling [22]. Here, we find evidence that a 2∶1 pattern could minimize work of respiratory muscles by allowing one footstrike to assist the ventilatory transition and the second to occur at intermediate lung volumes (see Fig. 6A). At intermediate lung volumes, the net effect of step-driven flows is either assistive or neutral (Fig. 4). However, near the end of each ventilatory half-cycle, approaching the extrema of lung volume, step-driven flows become antagonistic (Fig. 4). Higher coupling ratios require footstrikes near the end of each ventilatory half-cycle, leading to antagonistic loading of the respiratory system and transient reversals in flow (Fig. 3). Thus, a 2∶1 stride per breath rhythm might be more strongly preferred during intense endurance running, when fatigue of respiratory muscles could be limiting.

We hypothesize that human runners benefit from locomotor-ventilatory entrainment by reducing the work of ventilatory muscles, and minimizing fatigue of respiratory muscles that are critical to endurance aerobic activity. Appropriate tuning of locomotor-ventilatory interactions likely minimizes antagonistic loading of ventilatory muscles and allows inertial displacement of the guts to passively assist the action of respiratory muscles. These mechanical interactions likely have the greatest impact on diaphragm performance because the abdominal viscera directly attach to this muscle. Work of breathing increases as a squared function of ventilatory demand, and breathing may account for up to 10–15% of energy demand in intense exercise [38], [39]. Although the properties of the mammalian diaphragm appear to confer considerable resistance to fatigue [40], some evidence suggests that it can be vulnerable to fatigue during prolonged or intense exercise in both sedentary and fit individuals [41], [42], [43], [44], [45], [46], [47]. Declines in respiratory function following marathon and ultra-marathon competitions also suggest respiratory muscle fatigue [48], [49], [50]. Respiratory muscle fatigue may be a limiting factor in human endurance activity, and locomotor-ventilatory coupling has potential to minimize fatigue, especially during activities that involve impact loading with each footstrike, such as walking and running.

Unfortunately, it remains challenging to directly test this hypothesis. Ventilatory muscles are a relatively small fraction of the metabolically active tissue in the body, making it difficult to measure changes in respiratory muscle work using standard respirometry techniques. Though technically challenging, it may be possible to use recordings of muscle activity (e.g., [51]) to examine the response of ventilatory muscles to manipulations of visceral load and locomotor-ventilatory coordination during running.

In future work, it would be interesting to investigate whether antagonistic locomotor-ventilatory interactions could explain, in part, why obese individuals experience ‘breathlessness’ and rapid fatigue during locomotion. Obese individuals face a dual problem of increased energy cost and impaired respiratory function during locomotion. Obese individuals incur a 10–25% higher metabolic energy cost of walking per kilogram body mass compared to people with healthy weight [52], [53]; meaning that carrying fat costs more than carrying lean weight. The source of this added cost remains controversial. External work does not explain the added cost, because the gait of obese individuals involves similar total mechanical work on their body center of mass [54]. Increased internal work associated bouncing soft tissues and increased costs for stabilizing the body and joints may contribute to increased locomotor costs due to excess fat mass [53], [54], [55], [56], [57], [58]. Furthermore, excess fat is associated with reduced operating lung volumes and decreased respiratory compliance, factors that increase the work of breathing [59], [60]. Soft-tissue bouncing induced by impact loads during walking and running may further increase the work of breathing and the rate of respiratory muscle fatigue in overweight and obese individuals. Whereas in lean individuals a large fraction of soft-tissue mass is concentrated intra-abdominally, obesity results in a large fraction of soft-tissue mass distributed external to the body wall muscles. Thus, high adiposity will increase soft-tissue bouncing and reduce the ability to actively tune soft-tissue dynamics through abdominal muscle contraction. Larger, uncontrolled soft-tissue motions may result in antagonistic locomotor-ventilatory interactions and higher mechanical work of breathing during exercise. We predict that the problems of obesity are exacerbated by antagonistic locomotor-ventilatory interactions during walking and running.

### Conclusions

Biomechanics studies often focus on musculoskeletal and biomechanical factors as limits to performance. For example, leg muscle strength is widely thought to limit top running speed during sprinting [61], [62]. However, during endurance locomotion, respiratory muscles, not leg muscles, may limit maximum exercise intensity and duration [47]. We suggest that when assessing the physiological importance of locomotor-respiratory coupling in humans, it may be short sighted to place too much emphasis on the comparatively small volumes attributed to it. Entrainment might serve a number of beneficial physiological functions – i.e., reducing the work of ventilatory muscles, preventing respiratory muscle fatigue, and improving respiratory efficiency through enhanced gas mixing, transport and exchange. Consequently, the precise volume driven by locomotion might be less important than the associated intrapulmonary dynamics. Humans are well adapted for economic walking and endurance running [34], [63], [64], [65]. Multiple derived characters suggest aerobic endurance as a key evolutionary pressure in the human lineage, including skeletal morphology, developed tendon springs and enhanced heat dissipation [34], [63], [64], [66]. We will not be surprised, therefore, if the unusual locomotor-respiratory coupling patterns exhibited by human runners proves to be another example of evolutionary adaptation in support of the exceptional endurance running capabilities of humans [63], [64].
